# Activated charcoal-mediated RNA extraction method for *Azadirachta indica* and plants highly rich in polyphenolics, polysaccharides and other complex secondary compounds

**DOI:** 10.1186/1756-0500-6-125

**Published:** 2013-03-28

**Authors:** Raja Rajakani, Lokesh Narnoliya, Neelam Singh Sangwan, Rajender Singh Sangwan, Vikrant Gupta

**Affiliations:** 1 Biotechnology Division, CSIR-Central Institute of Medicinal and Aromatic Plants, Lucknow, 226015, India; 2 Department of Metabolic and Structural Biology, CSIR-Central Institute of Medicinal and Aromatic Plants, Lucknow, 226015, India

**Keywords:** Activated charcoal, CTAB, Medicinal plants, Polysaccharides, RNA isolation, Secondary metabolite

## Abstract

**Background:**

High quality RNA is a primary requisite for numerous molecular biological applications but is difficult to isolate from several plants rich in polysaccharides, polyphenolics and other secondary metabolites. These compounds either bind with nucleic acids or often co-precipitate at the final step and many times cannot be removed by conventional methods and kits. Addition of vinyl-pyrollidone polymers in extraction buffer efficiently removes polyphenolics to some extent, but, it failed in case of *Azadirachta indica* and several other medicinal and aromatic plants.

**Findings:**

Here we report the use of adsorption property of activated charcoal (0.03%–0.1%) in RNA isolation procedures to remove complex secondary metabolites and polyphenolics to yield good quality RNA from *Azadirachta indica*. We tested and validated our modified RNA isolation method across 21 different plants including *Andrographis paniculata*, *Aloe vera*, *Rosa damascena*, *Pelargonium graveolens, Phyllanthus amarus* etc. from 13 other different families, many of which are considered as tough system for isolating RNA. The A260/280 ratio of the extracted RNA ranged between 1.8-2.0 and distinct 28S and 18S ribosomal RNA bands were observed in denaturing agarose gel electrophoresis. Analysis using Agilent 2100 Bioanalyzer revealed intact total RNA yield with very good RNA Integrity Number.

**Conclusions:**

The RNA isolated by our modified method was found to be of high quality and amenable for sensitive downstream molecular applications like subtractive library construction and RT-PCR. This modified RNA isolation procedure would aid and accelerate the biotechnological studies in complex medicinal and aromatic plants which are extremely rich in secondary metabolic compounds.

## Background

Medicinal plants are a big part of nature’s human protection against various diseases which attracted the researcher’s attention towards unveiling their bioactive molecules synthesis, whole biological pathway framework, transcriptome network and other molecular and cellular mechanisms. *Azadirachta indica* (family: *Meliaceae*) has been universally accepted as a nature’s tree of wonder for its diverse utility and omnipotent medicinal values. All the parts of *Azadirachta indica* like fruits, leaves, seeds, flowers, twigs, roots, gum, stem and bark have been used as a household remedies for various human ailments [1]. In addition, it has also been established as a potential source of naturally occurring insecticides, pesticides, anti-malarial agent and agrochemicals [2]. During the past couple of centuries, around 140 compounds have been isolated from different parts of *Azadirachta* tissues and tested in various *in vivo* models for its biological activity against a wide range of diseases like cancer, malaria and various fungal and bacterial infections. Azadirachtin, a major limonoid derivative which is well known for its anti-feedant and anti-malarial activity, is abundantly present in *Azadirachta* fruit endocarp along with other secondary metabolites such as gedunin, nimbin, meldin, isomeldin, mahmoodin and nimocinol. Other pharmaceutically important compounds like salanin and quercetin are majorly present in fruit mesocarp and leaf, respectively [3]. Biotechnological tools are now being exploited to identify and characterize the enzymes and genes underlying the metabolic pathways responsible for biosynthesis of these pharmaceutically important secondary compounds from *A. indica* for the benefit of mankind.

In most of the biotechnological and molecular investigation, a high quality RNA is the primary requisite for the downstream applications and tools, but, its extraction from many of the medicinal plants is tedious due to the presence of rich amount of polyphenols, polysaccharides, flavonoids, alkaloids, terpenoids, quinones and other diverse array of compounds. Since standard RNA extraction methods like guanidine thiocyanate phenol-chloroform method [4], modified hot borate method [5], and cetyltrimethylammonium bromide (CTAB) method [6] failed to yield appropriate quality and quantity of RNA from several plants rich in secondary metabolites and polysaccharides, therefore, modifications in the extraction procedures were attempted by several researchers to isolate good quality RNA for downstream applications. Their countermeasures for the removal of polyphenolics, polysaccharides and other complex secondary metabolites were the increase in the volume of extraction buffer in order to dilute the polyphenolics and the addition of vinyl-pyrollidone polymers (PVP or PVPP) in the extraction buffer [7,8]. High molecular weight polyethylene glycol (PEG) for the removal of polyphenolics compounds was also attempted [9]. Apart from these, addition of higher concentration of β-mercaptoethanol, a strong reducing agent, in the extraction buffer prevents the oxidation of phenols to quinones which covalently couple with nucleic acids [10]. Extraction method using water saturated phenol followed by acetic acid precipitation keeps the pH low (acidic) which increases the RNA stability [11], and, employing extraction buffers having high ionic strength in combination with lithium chloride precipitation effectively prevents the co-precipitation of polysaccharides along with nucleic acids [12]. These modifications in RNA extraction procedures also failed in case of diverse plant systems having complex secondary compounds. Recently, modified RNA isolation methods are reported for a few specific and tough medicinal plant systems that are rich in secondary metabolites [11,13]. Even modifications in the commercial kits have also been attempted to achieve a good quality of RNA from secondary metabolites-rich plants [14]. Still there is a need to develop and validate a method which could be employed for a wide range of complex plants belonging to different families. A few DNA extraction and purification methods from secondary metabolite-rich plant tissues are reported where activated charcoal with or without PVP was used for the efficient removal of polyphenols and secondary metabolites [15,16]. Activated carbon is a form of carbon which has been extensively used to remove various contaminants from water, soil and air with its adsorption capacity which has proved to be useful in pharmacy, industry and microbiology. It has also been successfully used in plant tissue culture to prevent growth inhibition due to excess polyphenolics which are released into the medium [17] as well as to remove PCR inhibitors and increase the sensitivity of real-time PCR reactions [18].

For implementing the biotechnological tools to study complex plants rich in secondary metabolites at the molecular level, there is a need for a unique RNA isolation procedure. We utilized the adsorption property of activated charcoal along with PVPP and varying concentrations of other components in the RNA extraction buffer for the effective removal of polyphenolics and other complex secondary metabolites from the RNA preparations isolated from *Azadirachta indica* which was further tested with diverse other complex medicinal and aromatic plants. Recently, the transcriptomes of *A. indica* was analyzed and reported which utilized commercially available kits for total RNA isolations [19,20]. Here in this report, we developed a simple, robust, and cost effective RNA extraction method which can be followed very easily in any molecular biology laboratory using general RNA isolation chemicals and activated charcoal. Commercially available kits usually yield limited amounts of total RNA and may sometimes need multiple extractions. Our developed method is advantageous over the commercial kits as it can be conveniently scaled up to yield bulk amount of total RNA and multiple extractions can be avoided. Our modified RNA extraction procedure yielded high quantity of RNA which was of extremely good quality, free from all sorts of contaminants including polysaccharides, polyphenolics and coloured secondary metabolic compounds, and, amenable for sensitive downstream biotechnological applications.

## Methods

### Plant materials

*Azadirachta indica* fruit and leaf tissues were plucked and snap frozen in liquid nitrogen from the tree planted at CSIR-National Botanical Research Institute, Lucknow, India. The frozen tissue was stored at -80°C until use. The leaf tissue of other selected plants, *Phyllanthus amarus*, *Aloe vera*, *Pelargonium graveolens*, *Rosa damascena*, *Andrographis paniculata*, *Artemisia annua*, *Catharanthus roseus*, *Ocimum basilicum*, *Ocimum gratissimum*, *Ocimum sanctum*, *Tephrosia purpurea*, *Withania somnifera*, *Merremia gangetica*, *Wedelia chinensis*, *Cannabis sativa*, *Scoparia dulcis*, *Mentha arvensis*, *Mentha piperita*, *Bacopa monnieri*, *Hemidesmus indicus* and *Glycyrrhiza glabra* that were used to validate the RNA extraction protocol were plucked from the research fields of CSIR-Central Institute of Medicinal and Aromatic plants, Lucknow, India and stored in a similar way.

### RNA extraction protocol

One gram of tissue was finely ground by using liquid nitrogen in a pre-chilled mortar and pestle. The powder was transferred immediately to a 10 ml of pre-warmed CTAB extraction buffer [2% w/v CTAB, 200 mM Tris-Cl (pH 8.0), 50 mM EDTA (pH 8.0) and 2.5 M NaCl] containing 0.03%-0.1% activated charcoal (AC), 1.5% PVPP and 3% β-mercaptoethanol in a sterile DEPC-treated tube. Activated charcoal, PVPP and β-mercaptoethanol are added in extraction buffer just before using it and vortexed and should be utilized immediately or within 3 days of its preparation. The activated charcoal remains uniformly suspended along with few floating particles after vortexing in the extraction buffer. The ground tissue powder in buffer mixture was vortexed vigorously for 1 min, and then incubated for 15 min in a water bath maintained at 65°C with intermittent vigorous shaking. The mixture was allowed to cool to room temperature (RT) and an equal volume of Chloroform/Isoamyl alcohol (24:1, v/v) mixture was added and mixed well by inverting the tubes. Mixture was then centrifuged at 12000 rpm for 20 min at 4°C, and the upper aqueous phase was carefully collected in a fresh sterile DEPC-treated tube. Almost all the charcoal particles along with the cell debris get pelleted at the bottom of the tube and removed along with the adsorbed complex secondary metabolites. Equal volume of water-saturated Phenol/Chloroform/Isoamyl alcohol (25:24:1, v/v) was added and mixed well, followed by centrifugation at 12000 rpm for 15 min at 4°C. The above extraction can be repeated once again to get a clear interface. Upper aqueous phase was then carefully collected (without disturbing the interface) in a fresh tube and an equal volume of Chloroform/Isoamyl alcohol (24:1, v/v) was added, mixed and centrifuged again at 12000 rpm for 15 min at 4°C. This step removes the residual phenol from the aqueous phase and avoids its traces in the final prep which otherwise hinders the downstream enzymatic applications. The upper aqueous phase was carefully collected in a fresh tube and one-fourth volume of 10 M lithium chloride was added, thoroughly mixed and incubated overnight at 4°C. The pellet was collected by centrifugation at 13000 rpm for 15 min at 4°C followed by washing with 2 M lithium chloride. The pellet was then dissolved completely in a suitable amount (200-300 μl) of RNase-free water. To the above solution, 1/10 volume of 3 M sodium acetate and 2.5 volume of chilled absolute ethanol were added, mixed and incubated at -80°C for 30 min. The pellet was again collected by centrifugation at 13000 rpm for 15 min at 4°C and washed with 75% ethanol. Finally, the pellet was dried in a desiccator under vacuum to remove residual ethanol and was re-suspended in a minimum volume of RNase-free water. All the plastic and glass wares used were treated with 0.01% diethyl pyrocarbonate (DEPC) and the solutions were prepared in MQ-water treated with 0.1% DEPC.

### Construction of subtractive hybridization library and RT-PCR

*A. indica* produce different secondary metabolic compounds of therapeutic and medicinal uses in an organ- or tissue-specific/abundant fashion. The fruit and leaf tissues of *A. indica* are rich in azadirachtin and quercetin, respectively, indicating a differential and tissue-specific expression of the concerned pathway genes. Therefore, in order to identify key genes of these secondary metabolic pathways and to understand their differential activities, we attempted to construct subtractive cDNA libraries from these tissues of *A. indica*. A good quality total RNA was isolated from young leaf and immature fruit of *Azadirachta indica* and subsequently used for purifying mRNA by using Oligo-dT cellulose (Sigma Aldrich, USA). Construction of subtractive library was carried out by using Clontech PCR-Select™ cDNA Subtractive Kit (Clontech, USA) following the manufacturer’s instructions. In forward subtraction, immature fruit and young leaf were taken as tester and driver, respectively, and *vice versa* for the reverse subtraction. *Rsa*I digested double stranded subtracted cDNA was amplified by primary and secondary PCR using adaptor and nested PCR primers provided by the manufacturer for the enrichment of tissue-specific genes. The amplified secondary PCR products were ligated in pGEMT-Easy vector system (Promega, USA) and transformed in *E. coli* DH10B electro-competent cells (Invitrogen, USA) by electroporation. Plasmids were isolated from few randomly selected clones by using standard alkaline lysis method and restricted by *Eco*RI restriction enzyme (Fermentas, USA) to check the presence of insert. Sequencing of the plasmids were done by using BigDye terminator v3.0 cycle sequencing kit (Applied Biosystems, USA) as per manufacturer’s instruction and analyzed in ABI Prism 3130XL Genetic Analyzer (Applied Biosystems, USA).

In our in-house generated EST sequences of *A. indica*, a homolog of cytochrome P450 was identified. Gene-specific forward primers (Forward primer 1: CAATCGTGCTGTTGAAGGGTCGAG and Forward primer 2: TGTCGCAAGATATTCGTGGACATC) for nested PCR were designed from the 5^′^ end of the EST and synthesized to carry out PCR amplification. Oligo-dT primer was used as a reverse primer for carrying out RT-PCR. The reverse transcription reaction for converting the RNA isolated by our modified method to cDNA was done using the ThermoScript RT-PCR system (Invitrogen, USA) as per the user manual. Primary PCR was carried out using forward primer 1 and oligo-dT primers. Using the primary PCR product as a template, secondary PCR was done using forward primer 2 and oligo-dT primers. The PCR was carried out by using 0.3 units of *Taq* DNA polymerase (Invitrogen, USA) and a specific amplification product was noted which was cloned in pGEMT-Easy vector and confirmed by sequencing.

In order to check the DNA contamination in total RNA previously extracted by our method, PCR was carried out using 1 μg of total RNA preparation (without converting into cDNA) as a template and primers specific to *A. indica* EST homologs of *actin* (Forward primer: AGGCATCCACGAGACCACTT and Reverse primer: TGGCGCTAGAGCAGAAATTTC) and *elongation factor* (Forward primer: AACCAGTGTTCGTGAAGACCAA and Reverse primer: AAGCATTTCATCCGCCTCAT) which were identified in our EST pool. For positive control, we made cDNA by using Thermoscript RT-PCR system (Invitrogen, USA) from total RNA isolated from *A. indica* immature fruit and young leaf. These cDNAs were further used as templates for PCR using the same *actin* and *elongation factor* EST-specific primers. The primers were designed in such a way so that 151 bp fragment would be amplified in case of positive control.

## Results and discussion

**Figure 1:**
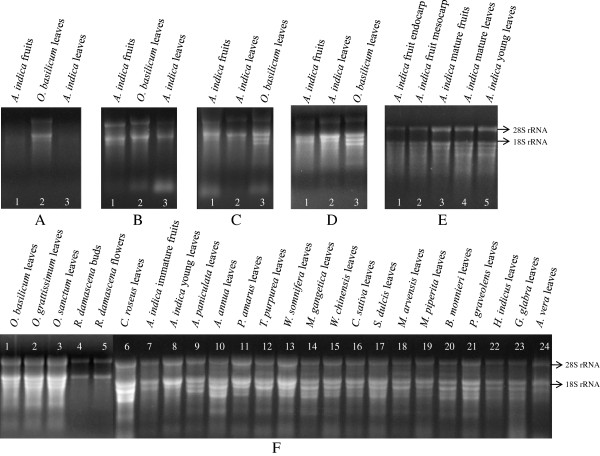
**Denaturing agarose gel electrophoresis of total RNA isolated from *A. indica *and other plant tissues using modified CTAB-based methods.** (**A**) Quality of total RNA isolated by using modified extraction buffer containing only PVPP and precipitation done by using glacial acetic acid. (**B**) RNA isolated by using modified extraction buffer containing only activated charcoal and precipitation done by using glacial acetic acid. (**C**) RNA extracted by using modified extraction buffer containing activated charcoal as well as PVPP followed by glacial acetic acid precipitation. (**D**) RNA extracted by using modified extraction buffer containing activated charcoal as well as PVPP followed by 2.5 M Lithium Chloride precipitation. (**E**, **F**) RNA extracted from different tissues of *Azadirachta indica* and other medicinal and aromatic plants by using our modified and optimized protocol.

The diverse array of tissue-specific pharmaceutically important secondary compounds produced in different tissues of *Azadirachta indica* attracted our attention to unveil their biosynthesis at the molecular level. In order to make a cDNA library, we tried to extract RNA from different tissues (young leaf, mature leaf, immature fruit, mature fruit, endocarp and mesocarp) of *Azadirachta indica* using standard Guanidine Isothiocyanate (GITC) and Guanidine Hydrochloride (GH) methods. GITC- and GH-based extraction methods are widely used in the model plants such as *Arabidopsis* and rice which gives a very good yield and quality of the RNA. The guanidine ion-based standard protocols did not yield any RNA from the tissues of *A. indica*. We then attempted the use of PVP or/and PVPP in the guanidine ion-based extraction buffers which makes complexes with polyphenolics through H-bonding, but could not get any RNA yield. After getting failed several times successively, we switched to CTAB-based extraction procedure [6] and modified version for medicinal plant seabuckthorn [11]. Commercially available RNA extraction kit from QIAGEN was also tried following the manufacturer’s instruction. No RNA could be obtained even when CTAB-based methods and kit were used. We believed that the failures in the RNA extraction from *A. indica* tissues by using standard procedures were because of the fact that the plant is highly rich in polyphenolics and other complex secondary metabolites. We altered the composition of extraction buffer to 200 mM Tris-Cl and 50 mM EDTA along with 1.5% PVPP which have strong H-receptor for binding and removal of polyphenolics [8] and 3-4% β-mercaptoethanol (a higher concentration) to provide highly reducing environment to block the oxidation of polyphenolics to quinones which is known to covalently complex with nucleic acids [10]. RNA yield and quality have been reported to be improved by increasing the ionic strength of the extraction buffer or by modifying the final washing steps [11]. Increase in the ionic strength of the extraction buffer by using high concentration of NaCl (2.5 M) followed by acetic acid precipitation or LiCl selective precipitation and washing with 2 M LiCl to remove polysaccharides in the final steps of the RNA extraction usually works with many of the tough plant systems [8,11] but it yielded extremely low amount and poor quality of total RNA from *A. indica* (Figure 1, Panel A). The difficulty in RNA extraction from *A. indica* tissues indicates that there might be a huge amount of polyphenolics and lot more of other complex secondary metabolites which could not be removed efficiently by using PVPP and probably hindered the RNA extraction.

Use of activated charcoal was reported in few of the DNA extraction procedures [15,16]. The carbon-based impurities like compounds containing chromogenic groups and large organic molecules are trapped and adsorbed reversibly by activated charcoal. In order to achieve RNA yield, we used the adsorption potential of activated charcoal (AC) to effectively remove the hampering secondary metabolites during RNA isolation process in the initial step itself because cytosol-borne secondary compounds including polyphenolics associate with AC and all the AC-trapped contaminants including particulate AC could be completely removed by initial centrifugation step. Inclusion of 0.03-0.1% AC in the extraction buffer could yield significant amount of total RNA from *A. indica* tissues and *O. basilicum* leaves (Figure 1, Panel B) which was further improved by the addition of PVPP along with activated charcoal (Figure 1, Panel C and D). The cytosol-borne secondary metabolic compounds associate efficiently with activated charcoal (AC) earlier than nucleic acids, and, PVPP also has a synergistic effect in binding polyphenols on AC due to the sp2-electronic configuration of carbon rings [15]. The above improvement in the extraction buffer and the differential precipitation using glacial acetic acid could extract good quality and quantity of RNA from *A. indica* and *O. basilicum* leaves (Figure 1, Panel C). Therefore, to develop a premium and modified RNA extraction method, we further tested the selective precipitation by using lithium chloride (LiCl) rather than acetic acid in our modified protocol. This change gave extremely good quality and quantity of total RNA from *O. basilicum* leaves and different tissues of *A. indica* (Figure 1, Panel D,E). Finally, an excellent and suitable modified protocol for RNA extraction from plants known to possess enormous amount of polyphenolics and complex secondary metabolites was developed and standardized, which was further employed and tested for a wide range of complex medicinal and aromatic plants belonging to 13 other diverse families and have pharmaceutical and industrial importance such as *R. damascena*, *A. paniculata*, *P. graveolens*, *T. purpurea*, *M. gangetica*, *W. chinensis*, *C. sativa*, *S. dulcis*, *B. monnieri* etc. (Table 1 and Figure 1, Panel F). Total RNA obtained from tissues of *A. indica* and other plant systems were spectrophotometrically quantified and analyzed for the possible contamination of proteins and polysaccharides by taking A260/280 and A260/230 ratios, respectively. The amount of total RNA yielded from the same amount of plant tissue varied from 10 ± 01 μg/g to 354 ± 36 μg/g amongst different tested plants, but overall RNA quantity was good with appropriate A260/280 and A260/230 ratios (Table 1), and, the quality of total RNA was also high as evident by the presence of distinct 28S and 18S rRNA without any degradation (Figure 1, Panel E,F). The protocol was repeated at least three times from separate sets of tissues and was found to be reproducible and accurate. This protocol could successfully isolate total RNA from *A. indica* mesocarp and *A. vera* tissues which are highly succulent in nature although the amount obtained was very low as compared to other plant tissues.

**Table 1:** Purity and yield of total RNA extracted from different plant tissues using our modified RNA extraction procedure

**S. no.**	**Family**	**Plant species**	**Tissue**	**Purity (A260/280)**	**Purity (A260/230)**	**Total yield (μg/g tissue)**
1	*Meliaceae*	*Azadirachta indica*	Immature fruit	1.87 ± 0.02	2.32 ± 0.15	112 ± 25
		*Azadirachta indica*	Mature fruit	1.99 ± 0.08	1.81 ± 0.03	341 ± 56
		*Azadirachta indica*	Young leaf	1.92 ± 0.04	2.25 ± 0.07	354 ± 36
		*Azadirachta indica*	Mature leaf	1.90 ± 0.09	1.77 ± 0.07	107 ± 70
		*Azadirachta indica*	Fruit endocarp	2.04 ± 0.07	2.15 ± 0.06	234 ± 31
		*Azadirachta indica*	Fruit mesocarp	2.02 ± 0.16	2.06 ± 0.03	45 ± 15
2	*Phyllanthaceae*	*Phyllanthus amarus*	Leaf	1.91 ± 0.06	2.24 ± 0.09	288 ± 21
3	*Xanthorrhoeaceae*	*Aloe vera*	Leaf	1.86 ± 0.05	2.14 ± 0.03	10 ± 1
4	*Geraniaceae*	*Pelargonium graveolens*	Leaf	1.80 ± 0.09	1.67 ± 0.06	100 ± 22
5	*Rosaceae*	*Rosa damascena*	Buds	1.79 ± 0.17	1.53 ± 0.63	14 ± 4
		*Rosa damascena*	Flowers	1.84 ± 0.14	1.75 ± 0.16	28 ± 11
6	*Acanthaceae*	*Andrographis paniculata*	Leaf	2.01 ± 0.11	1.87 ± 0.05	122 ± 18
7	*Asteraceae*	*Artemisia annua*	Leaf	1.95 ± 0.02	2.13 ± 0.10	213 ± 20
		*Wedelia chinensis*	Leaf	1.96 ± 0.07	2.36 ± 0.06	181 ± 58
8	*Fabaceae*	*Tephrosia purpurea*	Leaf	1.94 ± 0.04	2.26 ± 0.22	216 ± 33
		*Glycyrrhiza glabra*	Leaf	1.95 ± 0.16	2.44 ± 0.21	70 ± 27
9	*Convolvulaceae*	*Merremia gangetica*	Leaf	2.02 ± 0.17	2.43 ± 0.05	80 ± 7
10	*Lamiaceae*	*Ocimum basilicum*	Leaf	1.93 ± 0.04	1.97 ± 0.03	256 ± 33
		*Ocimum gratissimum*	Leaf	1.84 ± 0.02	1.92 ± 0.01	302 ± 33
		*Ocimum sanctum*	Leaf	1.86 ± 0.03	2.05 ± 0.06	203 ± 55
		*Mentha arvensis*	Leaf	1.93 ± 0.01	2.37 ± 0.10	177 ± 23
		*Mentha piperita*	Leaf	1.82 ± 0.13	2.20 ± 0.24	219 ± 53
11	*Apocynaceae*	*Catharanthus roseus*	Leaf	1.87 ± 0.10	2.22 ± 0.09	262 ± 23
		*Hemidesmus indicus*	Leaf	1.95 ± 0.12	2.46 ± 0.07	105 ± 35
12	*Solanaceae*	*Withania somnifera*	Leaf	1.92 ± 0.10	2.31 ± 0.10	291 ± 25
13	*Plantaginaceae*	*Scoparia dulcis*	Leaf	1.88 ± 0.09	2.14 ± 0.13	214 ± 28
		*Bacopa monnieri*	Leaf	1.94 ± 0.08	2.35 ± 0.25	90 ± 14
14	*Cannabaceae*	*Cannabis sativa*	Leaf	2.06 ± 0.06	2.28 ± 0.05	194 ± 64

The RNA yield was calculated based on the following formula: yield = A _260_ × 40 × 1,000 × volume (μl)/material weight (g). Each value indicates the mean of 3 (*n* = 3) replicates and standard error (±SE). The statistical analysis was performed by SPSS 11.5 software.

**Figure 2:**
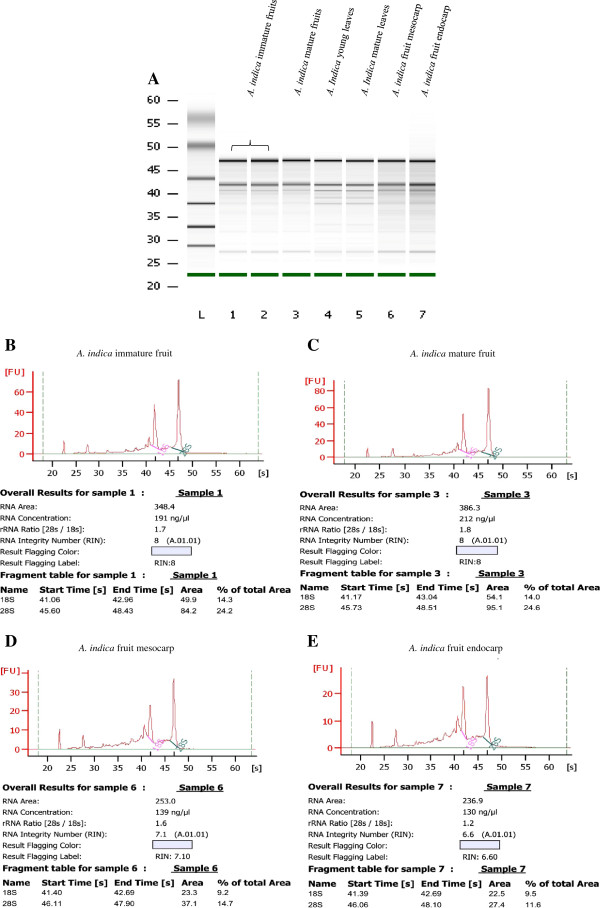
**Electrophoresis of total RNA prepared by our modified method from various *A. indica *tissues by using Agilent 2100 Bioanalyzer.** (**A**) Bioanalyzer gel image of small RNA ladder and *A. indica* tissues. (**B**-**E**) Bioanalyzer generated electropherograms of *A. indica* immature fruit, mature fruit, mesocarp and endocarp. The corresponding RNA Integrity Number (RIN) and ribosomal ratios are also indicated.

The integrity of the RNA is of paramount importance for several sensitive downstream applications. The integrity of total RNA was examined by the intensity and sharpness of rRNA bands visualized on denaturing 1.2% agarose gel (containing 3% formaldehyde) as well as in Agilent 2100 Bioanalyzer. The RNA sample for loading in the denaturing agarose gel was prepared in premix [20× MOPS/Formaldehyde/Formamide in the ratio of 1:3.5:10]. Heat shock were given to all sample mixtures at 65°C for 5 minutes and snap-chilled on ice for 5 min to melt any secondary structure. These samples were mixed with loading dye and run at 5 V/cm in 1× MOPS electrophoresis buffer. The gel was stained with EtBr and visualized under UV transilluminator which showed sharp and distinct bands and, the ratio of the brightness between 28S and 18S rRNAs were nearly 2:1 in most of the samples and 1:1 in some samples (Figure 1, Panel D-F). Next, the intactness and quality of total RNA prepared by our method was also analyzed by using Agilent RNA 6000 Nano Kit in an Agilent 2100 Bioanalyzer. The RNA Integrity Number (RIN values), electropherograms and gel-like images generated by Agilent 2100 Bioanalyzer indicated that the total RNA isolated from *A. indica* tissues was of acceptable quality (Figure 2). We analyzed the purity of RNA preparations from *A. indica* tissues spectrophotometrically by taking A260/280 and A260/230 ratios which ranged from 1.87 ± 0.02 to 2.04 ± 0.07 and 1.77 ± 0.07 to 2.32 ± 0.15, respectively (Table 1). Moreover, the Agilent 2100 Bioanalyzer generated RIN values also ranged from 6.6 to 8.0 (Figure 2) which further indicated an acceptable quality of total RNA for sensitive downstream applications.

**Figure 3:**
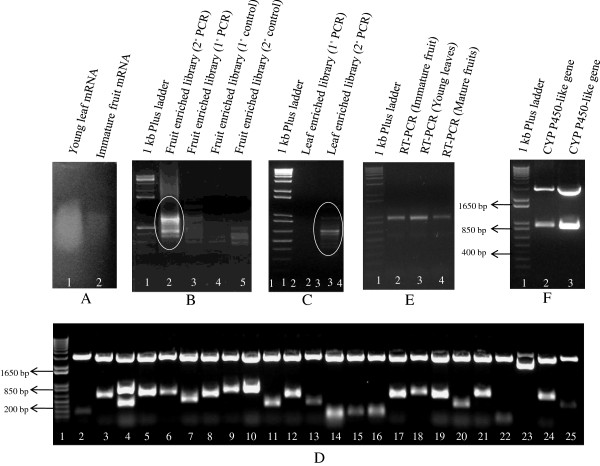
**Construction of subtractive cDNA libraries and RT-PCR. (A)** Purification of mRNA from *A. indica* tissues. (**B**, **C**) Primary and secondary PCR for immature fruit-enriched (**B**) and leaf-enriched (**C**) subtractive cDNA library. (**D**) Restriction digestion of plasmids isolated randomly from immature fruit-enriched subtractive cDNA library. (**E**) RT-PCR of *cytochrome P450-like* gene (a candidate in our library) by using the cDNA made from the total RNA isolated from different tissues of *A. indica* by our modified method. (**F**) Restriction digestion releasing the *cytochrome P450-like* gene fragment from the TA cloning vector (pGEMT-Easy) confirming its successful cloning.

Steps involved in reverse transcription and the construction of subtractive cDNA libraries are very sensitive to the impurities in the starting material (RNA) hence it is mandatory to isolate a high quality RNA for successful construction of libraries. In order to decipher genes taking part in tissue-specific secondary metabolism, subtractive cDNA libraries using young leaf and immature fruit tissue of *A. indica* were prepared using the RNA extracted by modified methodology standardized by us. The mRNAs were purified from the total RNA thus isolated and used to construct two separate subtractive cDNA libraries in which mRNA from immature fruit was taken as tester and young leaf mRNA as driver and *vice versa* (Figure 3, Panel A). The subtracted cDNAs were subsequently amplified by primary and secondary PCR (Figure 3, Panel B,C), ligated in TA cloning vector, and transformed in *E. coli* (DH10B) cells by electroporation. The quality of the libraries was checked by randomly picking the clones and analyzing the presence of insert by restriction digestions (Figure 3, Panel D). More than 95% of the plasmids had an insert successfully cloned. We randomly sequenced few ESTs and the sequence reads were found to be of good quality. A snapshot of the electropherogram of a representative EST is shown in Additional file 1 and few putative genes related to secondary metabolic pathways are indicated in Additional file 2.

Reverse transcription PCR is also one of the mainly used tools to amplify the gene of interest for further downstream usage. We used total RNA isolated from different *A. indica* tissues (young leaf, immature and mature fruit) by using our established protocol to reverse transcribe. To validate the quality of the cDNA prepared, we PCR amplified a representative partial *Cytochrome P450-like* gene (GenBank- JK126235), a candidate in our EST pool, using the cDNA as template. Specific and sharp PCR amplification indicated that the cDNA prepared was of a good quality (Figure 3, panel E). The partial fragment (874 bp) thus amplified was successfully cloned in TA cloning vector which was further confirmed by restriction digestion and sequencing.

In order to check the DNA contamination in our total RNA preparation, we PCR amplified partial fragments of *actin-like* gene (GenBank- JK624074) and *elongation factor-like* gene (GenBank- JK624104), representative EST candidates in our subtractive library pool, using total RNA (without RT) and cDNA (positive control) as templates. A specific and sharp PCR product for a partial fragment of *actin-like* gene and *elongation factor-like* gene was noted when cDNA was used as a template while no amplification was seen in the case of total RNA (without RT). This indicated that the total RNA prepared by using our modified method was free from any sort of DNA contamination and accessible for sensitive downstream applications (Additional file 3).

## Conclusions

We propose a modified protocol for isolating premium quality total RNA from *Azadirachta indica* which is a medicinal plant and contain abundant amounts of tannins, polysaccharides, polyphenolics and other secondary metabolic compounds. This developed protocol also worked well with other complex plant systems belonging to diverse families in which other common and modified methods usually fail. We employed the use of activated charcoal for the first time in RNA isolation along with PVPP in the CTAB-based modified buffer that proved successful. High ionic strength of the extraction buffer, selective precipitation and improved washing steps avoided polysaccharides to co-precipitate along with RNA. RNA obtained by this modified protocol was of high quality and suitable for downstream molecular and enzymatic applications which are otherwise sensitive to the contaminants.

## Abbreviations

β­M.E: β-mercaptoethanol; CTAB: Cetyl trimethyl ammonium bromide; GITC: Guanidine isothiocyanate; PVP: Polyvinyl pyrrolidone; PVPP: Polyvinyl polypyrrolidone; RT-PCR: Reverse transcription polymerase chain reaction.

## Competing interests

The authors declare that they have no competing interests.

## Authors’ contributions

RR and LN standardized various solutions and optimized protocol and carried out experiments. NSS and RSS guided the experimental designs. VG guided on development of the RNA extraction protocol and finally drafted the manuscript. All the authors have read the manuscript and agree with the content.

## Supplementary Material

Additional file 1 **A snapshot of the electropherogram of an EST (*cytochrome P450-like*) obtained in our subtractive cDNA libraries which were made by using our activated charcoal-mediated modified RNA extraction method.** Click here for file

Additional file 2 **A list of few representative EST clones in subtractive cDNA libraries made from *A. indica.*** Click here for file

Additional file 3 **PCR to check any DNA contamination in the total RNA preparations isolated by using our modified RNA extraction method. ***A. indica actin-like* and *elongation factor-like* genes which were identified in our EST pool were amplified using cDNA (positive control; Lanes 2-5) and total RNA (without RT; Lanes 6-9) from immature fruit and young leaves as templates. Click here for file
