# Supplementary figures and images for: Activated charcoal-mediated RNA extraction method for Azadirachta indica and plants highly rich in polyphenolics, polysaccharides and other complex secondary compounds
Source: BMC Res Notes. 2013 Mar 28;6:125. doi: 10.1186/1756-0500-6-125 (PMC3626780; doi:10.1186/1756-0500-6-125)

## Slide 1
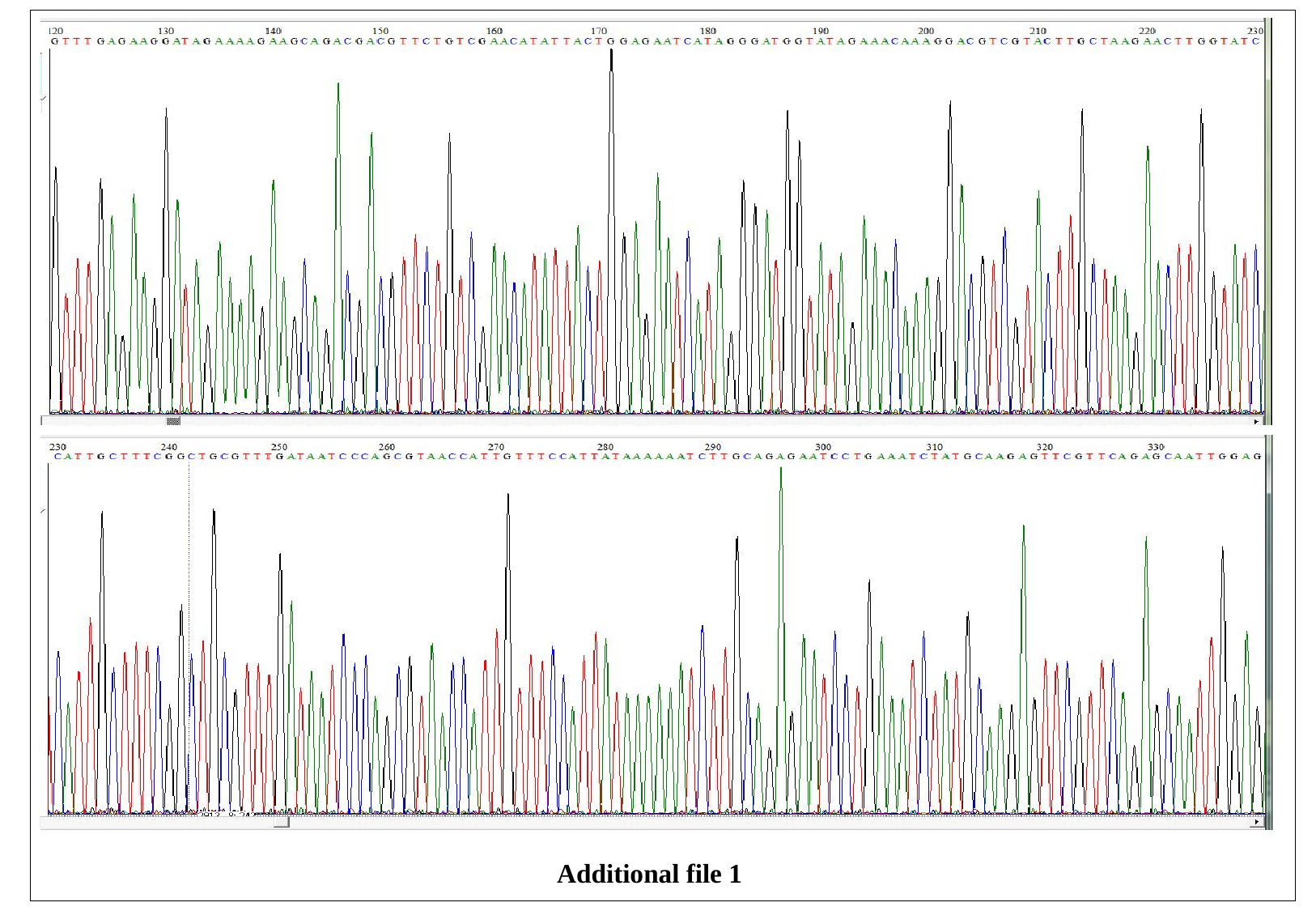

Additional file 1

Supplement: Additional file 1 — A snapshot of the electropherogram of an EST (cytochrome P450-like) obtained in our subtractive cDNA libraries which were made by using our activated charcoal-mediated modified RNA extraction method. [file 1756-0500-6-125-S1.ppt]
