# Supplementary material for: Activated charcoal-mediated RNA extraction method for Azadirachta indica and plants highly rich in polyphenolics, polysaccharides and other complex secondary compounds
Source: BMC Res Notes. 2013 Mar 28;6:125. doi: 10.1186/1756-0500-6-125 (PMC3626780; doi:10.1186/1756-0500-6-125)
Supplement: Additional file 2 — A list of few representative EST clones in subtractive cDNA libraries made from A. indica. [file 1756-0500-6-125-S2.doc]

**Additional file 2**

**Table 1 A list of few representative clones in subtractive cDNA libraries made from *A. indica.***

|  |
| --- |

**S. No. Clone Id. Accession Best match in NCBI database (BLASTx^b^) E-value^c^ Sequence**

**numbera identity (%)d**

|  |
| --- |

1 SclNMS-004-B09.b JK480072 NAC domain transcription factor [*Citrus trifoliata*] 2e-05 26/41 (63%)

|  |
| --- |

**2 SclNMS-001-A02.b JK126226 ARF domain class transcription factor [*Malus x domestica*] 1e-27 60/75 (80%)**

|  |
| --- |

3 SclNMS-001-G08.b JK341318 cyp-like protein [*Mytilus trossulus*] 3e-14 32/35 (91%)

|  |
| --- |

4 SclNMS-003-G01.b JK479930 CYP81B36 [*Scoparia dulcis*] 8e-28 55/109 (51%)

|  |
| --- |

5 SclNMS-001-C03.b JK126235 Putative taxadiene 5-alpha-hydroxylase cyp P450 [*Artemisia annua*] 4e-16 36/59 (61%)

|  |
| --- |

6 SclNMS-002-B01.b JK341348 Putative calcium-dependent protein kinase, [*Ricinus communis*] 2e-17 39/45 (87%)

|  |
| --- |

7 SclNML-001-C01.b JK998956 cytochrome P450 like-TBP [*Nicotiana tabacum*] 8e-50 107/155 (69%)

|  |
| --- |

8 SclNML-001-F01.b JK998959 cinnamoyl-CoA reductase, [*Ricinus communis*] 8e-40 66/87 (76%)

|  |
| --- |

9 SclNML-001-D04.b JK998981 anthocyanidin reductase [*Camellia sinensis*] 1e-117 170/203 (84%)

|  |
| --- |

10 SclNML-001-G04.b JK998984 dihydroflavonol 4-reductase [*Gossypium hirsutum*] 8e-72 98/119 (82%)

|  |
| --- |

11 SclNML-001-E09.b JK999022 anthocyanidin synthase [*Litchi chinensis*] 4e-128 180/197 (91%)

|  |
| --- |

**12 SclNML-003-B03.b JK999163 NADH-cytochrome b5 reductase-like protein [*Oryza sativa Indica*] 4e-19 39/44 (89%)**

| ^a^GenBank accession number, clones 1 to 6 are ESTs represented in *A. indica* subtracted cDNA library of immature fruit tissue and clones 7 to 12 are ESTs represented in *A. indica* subtracted cDNA library of leaf tissue.  ^b^Best BLASTx hit in the NCBI reference sequence database (BLOSUM 62%). The name of the plant in which similarity was found is indicated in parenthesis.  ^c^The expect (E) value refers to the number of matches expected by chance alone. The lower E value indicates a stronger supported match.  ^d^The amino acid identity. |
| --- |
