# Supplementary material for: Activated charcoal-mediated RNA extraction method for Azadirachta indica and plants highly rich in polyphenolics, polysaccharides and other complex secondary compounds
Source: BMC Res Notes. 2013 Mar 28;6:125. doi: 10.1186/1756-0500-6-125 (PMC3626780; doi:10.1186/1756-0500-6-125)
Supplement: Additional file 3 — PCR to check any DNA contamination in the total RNA preparations isolated by using our modified RNA extraction method. A. indica actin-like and elongation factor-like genes which were identified in our EST pool were amplified using cDNA (positive control; Lanes 2-5) and total RNA (without RT; Lanes 6-9) from immature fruit and young leaves as templates. [file 1756-0500-6-125-S3.ppt]

## Slide 1
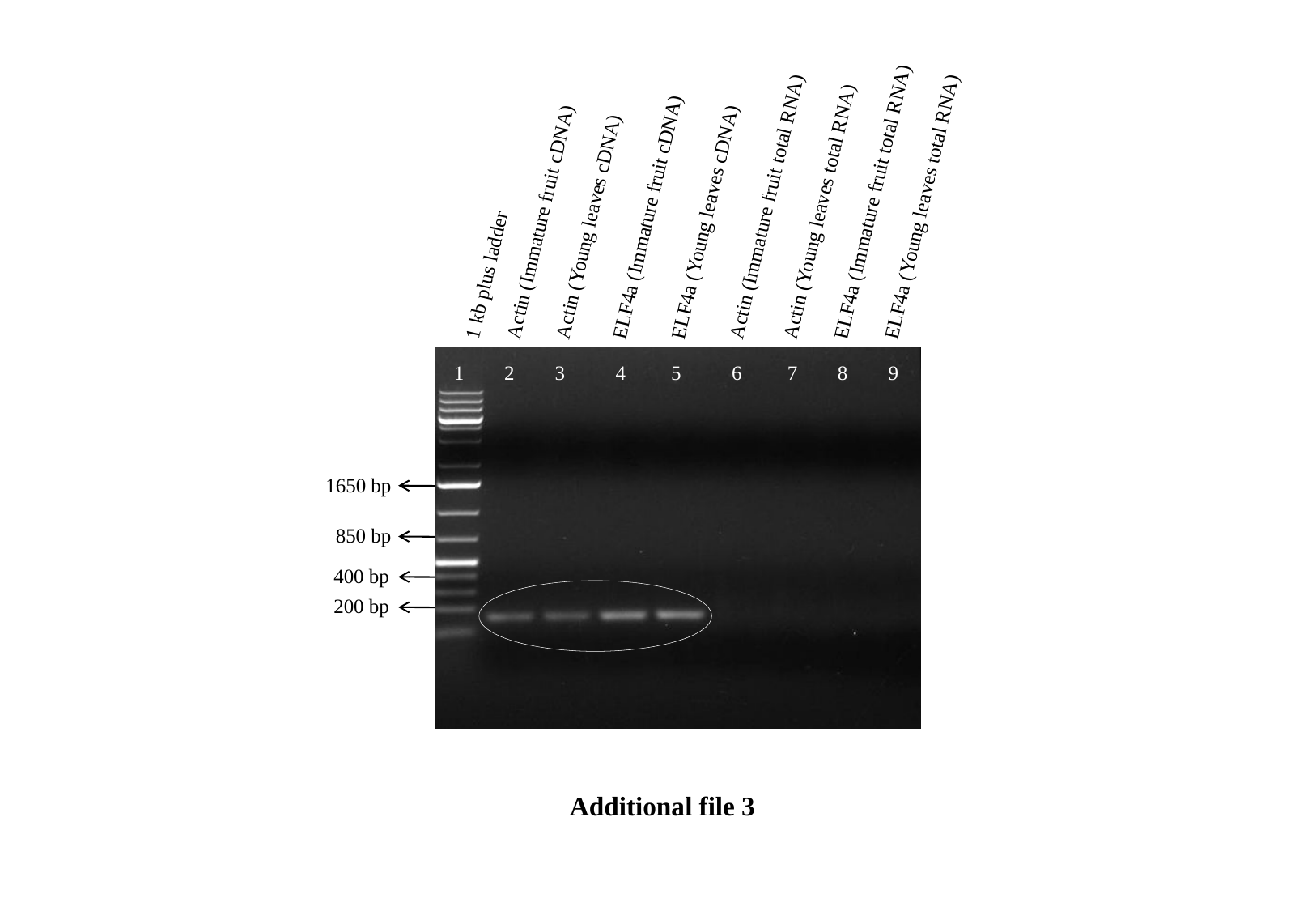

ELF4a (Immature fruit total RNA)
ELF4a (Young leaves total RNA)
Actin (Young leaves total RNA)
Actin (Immature fruit total RNA)
ELF4a (Young leaves cDNA)
ELF4a (Immature fruit cDNA)
Actin (Immature fruit cDNA)
Actin (Young leaves cDNA)
1 kb plus ladder
 1 2 3 4 5 6 7 8 9
1650 bp
850 bp
400 bp
200 bp
Additional file 3
